# Seroprevalence of swine hepatitis E virus and the farmers’ potential risk of infection in the Province of Bali, Indonesia

**DOI:** 10.14202/vetworld.2024.1810-1820

**Published:** 2024-08-20

**Authors:** I Made Kardena, Anak Agung Gde Oka Dharmayudha, I Wayan Nico Fajar Gunawan, Putu Devi Jayanti, I Nyoman Mantik Astawa, Anak Agung Ayu Mirah Adi, I Nyoman Suarsana, I Nyoman Suartha, Alan P. Dargantes

**Affiliations:** 1 Department of Pathobiology, Faculty of Veterinary Medicine, Udayana University, Jalan PB Sudirman, Denpasar, Bali, 80234, Indonesia; 2 Department of Veterinary Clinic, Faculty of Veterinary Medicine, Udayana University, Jalan PB Sudirman, Denpasar, Bali, 80234, Indonesia; 3 Department of Veterinary Basic Science, Faculty of Veterinary Medicine, Udayana University, Jalan PB Sudirman, Denpasar, Bali, 80234, Indonesia; 4 Department of Medicine, Surgery & Zootechnics, College of Veterinary Medicine, Central Mindanao University, Musuan, Maramag, Bukidnon, 8714, The Philippines

**Keywords:** Bali, hepatitis E virus, pigs, risk factors, seroprevalence

## Abstract

**Background and Aims:**

Hepatitis E virus (HEV) infection formerly and predominantly occurred in rural areas. However, it has recently been spread to urban and peri-urban areas. This study aimed to estimate the seroprevalence of HEV in pigs collected from urban and rural areas in Bali. The potential of the pig farmers’ risk level for being exposed to HEV and the virus transmitted to them in association with their pig-rearing practices was also assessed.

**Materials and Methods:**

A total of 183 pigs from 68 herds were sampled in this study, with 91 pigs collected from Denpasar as the representative samples of urban areas and 92 pigs from Karangasem Regency as the representative samples from rural areas. Sera from the sampled pigs were collected and immunoglobulin G antibodies against HEV were detected using a commercial enzyme-linked immunosorbent assay. A questionnaire was prepared for interviewing the farmers. Bivariable and multivariable logistic regression analyses were performed to identify the putative factors associated with seropositivity. Meanwhile, the potential risk-incurring practices of the farmers for HEV being transmitted to them from their pig-rearing practices were assessed by scoring their responses from the interview.

**Results:**

Overall, 23.5% (43/183) (95% confidence interval [CI]: 17.6–30.3) pig sera tested were detected to have the antibodies against HEV. Among 68 pig herds, 36.8% (25) (95% CI: 25.4–49.3) of them had antibodies in at least one pig sampled from each herd. Pigs sampled from Karangasem were 5 times (Odds ratio [OR] 5.34, 95% CI: 2.27–13.54, p < 0.001) more likely to be seropositive than pigs collected from Denpasar. However, no difference was found in the seropositivity to HEV in pig herds between Denpasar and Karangasem (p = 0.05). In assessing the pig rearing management factors, pig farmers from Denpasar were 3 times (OR 3.0, 95% CI: 1.07–8.52, p = 0.05) more likely to rear pigs for economic investment compared to the farmers from Karangasem. Regarding anticipating pig diseases that can be transmitted to humans, farmers from Denpasar were 6 times (OR 5.72, 95% CI: 1.48–26.7, p = 0.0074) more likely to anticipate zoonotic diseases compared to the farmers from Karangasem. Similarly, pig farmers from Denpasar were 3 times (OR 3.29, 95% CI: 1.08–10.23, p = 0.035) more likely to anticipate pig diseases that could be transmitted to humans than the farmers from Karangasem. Pig farmers from Denpasar had 4 times the odds (OR 4.49, 95% CI: 1.11–18.19, p = 0.03) of washing their hands after going to the pigpens compared to the farmers from Karangasem. All the participants were categorized as being at high risk of HEV exposure and transmission.

**Conclusion:**

IgG antibodies against HEV were detected among pigs reared in rural areas of Karangasem and those reared in urban areas of Denpasar. This suggests that the risk of HEV exposure and transmission in these areas is not negligible. To minimize the risk, public education on zoonotic diseases, including HEV infection, transmission, and prevention, needs to be implemented and particularly targeted to local pig farmers.

## Introduction

Hepatitis E virus (HEV) is a zoonotic disease caused by HEV infection that can pass from animals to humans. This viral infection has already been spread globally. Twenty million HEV infections in humans are thought to occur annually around the globe, resulting in about 3.3 million clinical cases and more than 410,000 fatalities [1]. Among other genotypes, the HEV genotypes 3 and 4 infections are zoonotic and transmitted to people through contact with the infected pigs or by consuming undercooked pork or organs of the infected pigs. The infection may cause acute to chronic hepatitis [2] with relatively high morbidity and mortality, especially in pregnant women in developing countries [3]. The disease was initially dominant in rural areas where agricultural livestock is widely available [4]. Recently, it has been reported to be spreading into urban areas as well, and it is suspected of being spread by HEV-contaminated food made from pork-related products [5]. Food or pig products contaminated with HEV are reported as contributing to the high number of HEV cases in humans [5]. In developing countries, HEV infection is mostly a waterborne illness linked to widespread outbreaks brought on by contaminated water and water supplies [6], particularly in rural areas. However, detection of HEV infection in urban areas was reported in China, where dogs and cats in some developed cities of Beijing, Shanghai, Canton, Shenzhen, and Macao were surveyed and detected to have antibodies to HEV at 21.12% (139/658) and 6.28% (12/191), respectively [7]. In addition, close contact between the community and the pigs is a risk factor associated with seropositivity to HEV antibodies in humans. Pigs are considered the reservoir hosts of the zoonotic HEV [8] and pig farming practices are the main risk factors associated with this viral infection [9]. However, there is limited data available in regard to HEV infection in pigs, including their rearing management practices that are associated with HEV infection in the animals, especially in places where the density of pig population and demand for pork are high.

Indonesia is endemic to HEV infection [10], including the province of Bali. However, studies on the viral infection in pigs in Bali are very limited. Related reports that have already been performed so far are inadequate to fill the gaps in understanding the disease dynamics. The last reports on HEV infection in humans were recorded at 20% (55/276) [11] and 18% (241/2,450) of the antibodies being detected in pregnant women in Bali [12]. While in the animals, 72% (51/71) of antibodies to HEV were detected in Badung Regency in the province [11]. The prevalence increased to more than 80% (95/119) of the pig population collected from Mengwi subdistrict, a peri-urban area of Badung [13]. However, no data on the disease frequency has been reported from urban and rural areas on the island. In fact, the sociocultural activities of the Balinese likely lead to the high risk of HEV-related human cases in Bali. Many locals are rearing pigs not only for investment but also for traditional ceremonial activities [14], leading to a high density of pig population with an estimated proportion of pigs to humans of 1:4 [15]. This condition also likely contributes to close contact between pigs and the local community, especially pig farmers, and leads to a higher risk of being exposed to the virus and having the virus transmitted to them.

A study conducted by Wibawa *et al*. [16] revealed that the genetic sequence of HEV isolated from local human cases had a high similarity of 97.3%–98.3% to the swine isolates in Bali, indicating pigs are the reservoirs of HEV infection among the Balinese. In addition, 18.8% (12/64) of local swine farm workers or owners in Bali were detected to have HEV antibodies [13], indicating they might have limited information about the disease. Consequently, HEV transmission into Balinese is suspected to be through zoonotic transmission from pigs [12]. Given that the pig population and density are relatively high on the island, the frequency of the HEV infection in pigs and the risk factors associated with its occurrence need to be assessed.

This study aimed to estimate the seroprevalence of HEV infection in pigs from urban and rural areas and to identify the risk factors associated with the seropositivity of HEV antibodies in the areas of Bali Island. The potential risk level of the pig farmers being exposed to HEV and having the virus transmitted to them was also assessed.

## Materials and Methods

### Ethics approval and informed consent

This research protocol was approved by the Ethics Committee of the Faculty of Veterinary Medicine, Udayana University, with certificate number B/164/UN14.2.9/PT.01.04/2023. Before the blood was collected and the interview was performed, verbal consent about the purpose of the research, the reasons for the research conducted, and the target achievement were explained to the participants. They were also advised that being the participants in this research was voluntary, and they could withdraw their participation at any point.

After the consent was approved, sampled pigs belonging to the participants were bled. Then, the participants were interviewed using a standardized questionnaire to obtain information on their pig-rearing management practices.

### Study period and location

**Figure-1:**
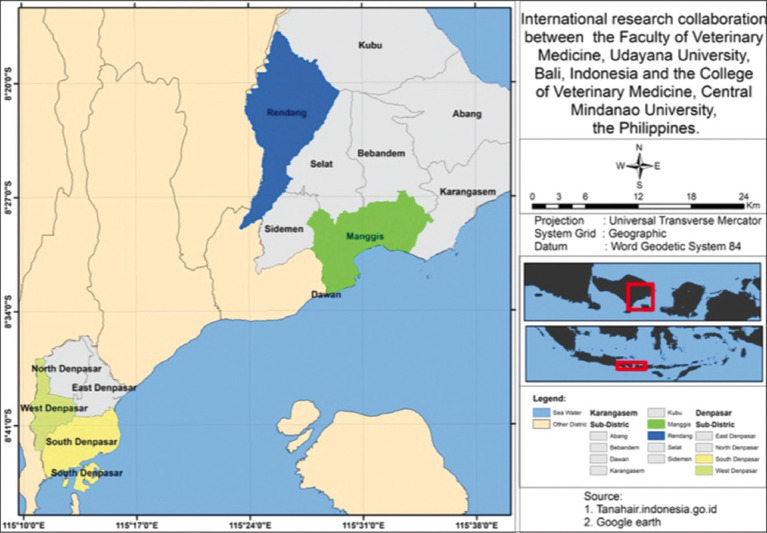
Map of the pig sampling areas in Denpasar City (subdistricts of South and West Denpasar) and Karangasem Regency (Subdistricts of Rendang and Manggis) on Bali Island, Indonesia.

A cross-sectional study was conducted from September to November 2023 in the province of Bali. Two district-level areas were included in this study, such as Denpasar City, which represents urban areas, and Karangasem Regency, which represents rural areas. Two subdistricts of the city and regency were randomly sampled. Two of the four subdistricts were sampled from Denpasar (South Denpasar and West Denpasar), whereas another two subdistricts of Karangasem (Manggis and Rendang) were also included (Figure-1).

### Sample size determination

The online software Epitools was used to calculate the minimal sample size of the pig samples. To detect a difference between the seroprevalence of HEV infection in pigs collected from urban and rural areas in Bali, an estimation of 20% of the different proportions between the areas was used with a prevalence of 0.815 [13], a sample size ratio of 1, a power of 0.8, and a confidence level of 95%. The total minimal sample pigs required was 178 for the two areas.

### Sampling on pig farmers and their pigs

Convenient sampling was used to choose the pig farmers or owners based on the lists obtained from the subdistrict level of Pusat Kesehatan Hewan (the Animal Health Posts) of the Dinas Pertanian (Department of Agriculture) from each of the subdistricts. The pigs were sampled based on the pig farmers’ or owners’ approval. Two to three pigs from each sampled pig herd were bled to get their sera.

### Pig farmers’ or owners’ interview

A questionnaire was prepared for an interview to get related information on their pig-rearing management practices and the risk of being exposed to HEV and having it transmitted to them. The contents of the questionnaire were divided into three sections, with a total of 33 questions included. The first section asked about individual animal factors, such as sex, age, vaccination status, and breed. The second section contained 19 questions asking about the participants’ pig-rearing management practices for herd-level factor identification, for example, their pig rearing system, purposes of rearing pigs, whether pigs were confined or not, number of pigs reared, frequency of cleaning the pens, feeding frequency, water given, etc. The third section contained 10 questions asking about the potential risk of exposure to HEV and having the virus transmitted to them in association with pig rearing practices of the pig farmer participants (Supplementary data).

### Sampled pigs’ blood collection

Two to three milliliters of the sampled pig blood were drawn from the anterior vena cava or jugular vein using a 3–5 mL syringe. After the collected blood clot and serum were released at room temperature, they were centrifuged at 1006 × *g* for 10 min before being kept in a −20°C freezer for a maximum of 3 months. One day before the samples were tested, they were taken from the freezer, allowed to defrost, and kept in a 4°C refrigerator.

### Enzyme-linked immunosorbent assay (ELISA) procedure and diagnostics

A commercial immunoglobulin (Ig) G ELISA kit (Wuhan Feiyue Biotechnology Co., Ltd., Wuhan, Hubei, China) was used to detect the antibodies against HEV in the sampled pig sera. The test was performed as per the manufacturer’s instructions, and the optical density of the test results was read at 450 nm wavelength.

### Statistical analysis

#### Individual-and herd-level seroprevalence

Individual animal and herd-level test seroprevalence and their 95% confidence intervals (95% CI) were calculated. Each of the prevalence figures was calculated based on the number of serum samples that tested positive for antibodies against HEV divided by the total number of serum samples tested. A herd was categorized as positive if one or more of the sampled pigs collected from the herd tested positive for the antibodies. The differences in seroprevalence between the two regions were analyzed with a Chi-square test for independence or Fisher’s exact test and the odds ratio (OR) with 95% CI and a p < 0.05 was considered as significant.

#### Risk factor identification in association with the seropositivity

Data on the participants’ pig-rearing management practices from both study areas were combined to identify the risk factors associated with seropositivity. The potential risk factors were assessed using univariable analysis and a Chi-square test using OpenEpi (https://www.openepi.com/). Factors that had an OR > 1.0 and a p ≤ 0.25 were included in a logistic regression model. The final model was built using backward elimination, with factors with a p < 0.05 were retained in the model. The fit of the data was assessed by evaluating the Hosmer–Lemeshow statistic and interaction between the reduced subset of variables assessed using IBM SPSS version 23 for Windows (IBM Corp., NY, USA).

#### Identifying the potential risk level of the participants in association with their pig-rearing practices

In determining the potential risk level of the farmers to HEV exposure and transmission in association with their pig-rearing practices, responses of the pig farmer participants to 10 questions in the third section of the questionnaire were used. Each answer to each question was scored “0” when the answer indicated had “low risk,” whereas the score was “1” when the response had “high risk.” The total score of all participants was calculated and categorized into two groups such as (i) low-risk practice, for the range score from zero to the cut-off value and (ii) high-risk practice, for having a range score higher than the cut-off value. The median of the total risk score was calculated and used for the threshold value of the low and high-risk levels. In differentiating the level of the risk practices between the participants surveyed from Denpasar and Karangasem, a Chi-square test was also performed and by calculating the OR with 95% CI and p < 0.05.

## Results

Overall, among the 183 total pigs sampled from both study areas, the seroprevalence obtained was 23.5% (43) (95% CI: 17.6–30.3). In 68 sampled pig herds, 36.8% (25) (95% CI: 25.4–49.3) of the herds were detected to have pigs with IgG antibodies against HEV.

In Denpasar, the individual seroprevalence was 9.9% (9/91) (95% CI: 4.6–17.9), and its herd prevalence was 23.5% (8/34) (95% CI: 10.7–41.2). However, the individual seroprevalence of the pig samples collected from Karangasem was higher at 37% (34/92) (95% CI: 27.1–47.7), and its herd-level seroprevalence was 50% (17/34) (95% CI: 32.4–67.6) (Table-1).

**Table-1:** Seroprevalence of HEV in pigs collected from Denpasar and Karangasem, Bali Province, Indonesia.

Areas		Positive samples		Total samples		Seroprevalence (95% CI)	
			
District level	Subdistrict level	Individual level	Herd level	Individual level	Herd level	Individual level	Herd level
Denpasar	South Denpasar	6	5	41	16	14.6% (5.6–29.2)	31.3% (11.0–58.7)
	West Denpasar	3	3	50	18	6.0% (1.3–16.5)	16.7% (3.6–41.1)
	Total	9	8	91	34	9.9% (4.6–17.9)	23.5% (10.7–41.2)
Karangasem	Manggis	18	9	45	17	40% (25.7–55.7)	52.9% (27.8–77.0)
	Rendang	16	8	47	17	34% (20.9–49.3)	47.1% (23.0–72.2)
	Total	34	17	92	34	37% (27.1–47.7)	50% (32.4–67.6)

CI=Confidence interval

Pigs collected from Karangasem were 5 times (OR 5.34, 95% CI: 2.27–13.54, p < 0.001) more likely to be seropositive to HEV compared to pigs collected from Denpasar. However, no difference was observed in the seroprevalence of HEV at the herd level between Denpasar and Karangasem (p = 0.05).

In identifying the risk factors associated with the seropositivity of the antibodies to HEV, results of the univariable analysis showed that vaccination status (OR 2.84, 95% CI: 1.33–6.13, p = 0.006) and age (OR 3.58, 95% CI: 1.65–7.89. p < 0.001) were the two potential individual-level risk factors associated with the seropositivity (Table-2).

**Table-2:** Individual-level potential risk factors associated with the seropositivity antibodies against HEV in pigs collected from Denpasar and Karangasem, Bali Province.

Determinants	ELISA results		Odds ratio		p-values
	
	Positive	Negative	OR	95% CI	
Vaccination status**					
Not vaccinated	26	49	2.84	1.33–6.13	0.0055*
Vaccinated	17	91			
Age					
≥6 months	28	48	3.58	1.65–7.89	<0.001*
<6 months	15	92			
Sex					
Female	28	93	0.94	0.44–2.09	>0.99
Male	15	47			
Breed***					
Landrace	20	82	0.62	0.29–1.30	0.22
Other breeds	23	58			

CI=Confidence interval, OR=Odds Ratio, ELISA=Enzyme-linked immunosorbent assay.

* p < 0.25 and OR > 1 were further analyzed using a logistic regression model,

** vaccination against hemorrhagic septicemia and/or classical swine fever,

*** breed: as specified by the farmers.

Meanwhile, univariable analysis identified potential herd-level factors, such as pig-rearing management system (OR 7.01, 95% CI: 2.01–24.4, p < 0.001), types of pigpen floor (OR 5.87, 95% CI: 1.76–19.9, p = 0.002), accumulated or still water found on the pen floor (on day of visit) (OR 4.1, 95% CI: 1.29–13.4, p = 0.014), cleanliness of the pigpens (on day of visit) (OR 3.11, 95% CI: 1.0–9.82, p = 0.051), dung or feces (sewage) around pigpens (OR 5.34, 95% CI: 1.77–16.16, p = 0.004), cleanliness of the feed containers (on day of visit) (OR 4.04, 95% CI: 1.35–12.11, p = 0.23), existence of other domestic animals around pigpens (OR 3.93, 95% CI: 0.92–23.38, p = 0.069), types of drinking water given (OR 5.28, 95% CI: 1.26–31.0, p = 0.018), existence of a natural water source around the pig herds (OR 4.4, 95% CI: 1.37–14.62, p = 0.0096), frequency of feeding (OR 2.81, 95% CI: 0.74–13.17, p = 0.16), and types of feed given (OR 3.49, 95% CI: 0.64–35.09, p = 0.2) (Table-3).

**Table-3:** Herd-level potential risk factors associated with the seropositivity of HEV in pigs collected from Denpasar and Karangasem, Bali Province.

Determinants	ELISA results		Odds ratio		p-value
	
	Positive	Negative	OR	95% CI	
Pig rearing management					
Extensive	17	10	7.01	2.07–24.44	< 0.001*
Semi-intensive	8	33			
Purposes for rearing pigs					
Investment	6	21	0.33	0.09–1.09	0.075
Others	19	22			
Pigs kept					
Confined/penned	20	5	0.65	0.15–3.07	0.74
Non-confined/non-penned	37	6			
Number of pigs reared					
≥10/pen	6	17	0.48	0.13–1.62	0.29
<10/pen	19	26			
Type of pigpen floor					
Non-cement (soil)	16	10	5.87	1.76–19.93	0.0021*
Cement	9	33			
Frequency cleaning pens					
>twice/day	4	17	0.29	0.06–1.10	0.0075
≤twice/day	21	26			
Accumulated water on the pen’s floor (on day visit)					
Yes	16	13	4.1	1.29–13.4	0.014*
No	9	30			
Cleanliness of pen (on day visit)					
Dirty	15	14	3.11	1.0–9.82	0.051*
Clean	10	29			
Dung (sewage)					
Piled	19	16	5.34	1.77–16.16	0.004*
Non-piled	6	27			
Dung usage					
Fertilizer	3	16	0.23	0.04–0.97	0.044
Biogas	22	27			
Cleanliness of feed container (on day visit)					
Dirty	12	8	4.04	1.35–12.11	0.23*
Clean	13	35			
Domestic animals around the pigpen					
Domestic animals around	22	28	3.93	0.92–23.38	0.069*
No domestic animals around	3	15			
Type of drinking water given					
Untreated water	22	25	5.28	1.26–31.0	0.018*
Treated water	3	18			
Natural water source around the herd (≤300 m)					
Yes	17	14	4.4	1.37–14.62	0.0096*
No	8	29			
Frequency of feeding per day					
≤twice/day	21	28	2.81	0.74–13.17	0.16*
>twice per day	4	15			
Types of feed given					
Concentrate	2	13	0.2	0.02–1.05	0.059
Mixed	23	30			
Feed processed before given					
Not cooked	23	33	3.49	0.64–35.09	0.2*
Cooked	2	10			
Feed and drink mixed in a container					
Mixed	8	29	0.22	0.07–0.73	0.096
Separated	17	14			
Pigs originated					
From outside village	13	20	1.25	0.42–3.75	0.85
From inside village	12	23			

* p-values < 0.25 and OR >1 were further analyzed using a logistic regression model. CI=Confidence interval, OR=Odds Ratio, ELISA=Enzyme-linked immunosorbent assay

Results of the logistic regression for identifying the putative individual-level risk factors showed pigs more than or equal to 6 months were 4 times (OR 3.9, 95% CI: 1.86–8.32, p < 0.001) more likely to be seropositive to HEV compared to those less than 6 months of age. Meanwhile, the putative risk factors in herd-level identified pigs reared in an extensive management system were 6 times (OR 5.9, 95% CI: 1.67–20.6, p < 0.01) more likely to be seropositive to HEV compared to the pigs reared in semi-intensive management. The existence of a natural water source around a pig herd (within 300 meters) was also associated with seropositivity. Pigs reared close to a natural water source, such as a river or bank and pond, were 6 times (OR 6.3, 95% CI: 1.70–22.9, p < 0.01) more likely to be seropositive to HEV compared to those pigs that were reared where the source of natural water farther away.

### Potential risk-level identification of the participants toward their pig-rearing practices in association with HEV exposure and transmission

One of the participants from Karangasem declined to take part in this survey, so only 33 participants from the area were included in the analysis (Table-4).

**Table-4:** Factor identification in association with the participants’ risk level in their pig-rearing practices toward HEV exposure and transmission.

Pig rearing practices	Denpasar		Karangasem		Total	
		
	Number	Percentage	Number	Percentage	Number	Percentage
Do you raise pigs using extensive systems?						
Yes	11	32.4	16	48.5	27	40.3
No	23	67.6	17	51.5	40	59.7
Is economic investment is the reason for keeping pigs?						
Yes	18	52.9	9	27.3	27	40.3
No	16	47.1	24	72.7	40	59.7
Do you prevent zoonotic diseases?						
Yes	15	44.1	4	12.1	19	28.4
No	19	55.9	29	87.9	48	71.6
Do you prevent pig diseases that can be transmitted to humans?						
Yes	20	58.8	10	30.3	30	44.8
No	14	41.2	23	69.7	37	55.2
Do you prevent jaundice disease?						
Yes	20	58.8	15	45.5	35	52.2
No	14	41.2	18	54.5	32	47.8
Has anyone in your family ever experienced jaundice?						
Yes	8	23.5	6	18.2	14	20.9
No	26	76.5	27	81.8	53	79.1
Do you live close to a pigsty (≤100 m)?						
Yes	23	67.6	24	72.7	47	70.1
No	11	32.4	9	27.3	20	29.9
Do you always wash your hands after going to the pig pen?						
Yes	31	91.2	24	72.7	55	82.1
No	3	8.8	9	27.3	12	17.9
Have you ever eaten undercooked pork?						
Yes	18	52.9	26	78.8	46	65.7
No	16	47.1	7	21.2	23	34.3
Have your family members ever eaten undercooked pork?						
Yes	24	70.6	27	81.8	51	76.1
No	10	29.4	6	18.2	16	23.9

HEV=Hepatitis E virus

No difference was found in reasons for rearing pigs between pig farmers from Denpasar and Karangasem (p = 0.05). In regard to anticipating pig diseases that can be transmitted to humans, the farmers from Denpasar were 6 times (OR 5.72, 95% CI: 1.48–26.65, p = 0.0074) more likely to prevent zoonotic diseases compared to the farmers from Karangasem. Similarly, the pig farmers from Denpasar were 3 times (OR 3.28, 95% CI: 1.08–10.23, p = 0.035) more likely to prevent pig diseases that could be transmitted to humans than the Karangasem farmers. Pig farmers from Denpasar had 4 times the odds (OR 4.49, 95% CI: 1.11–18.19, p = 0.03) of washing their hands after going to the pigpens compared to the farmers from Karangasem. However, although more than half of the total participants were categorized as potentially having a high-risk level, no significant difference was observed in the risk level between the participants from Denpasar and Karangasem (p = 0.27) (Table-5).

**Table-5:** The different levels of potential risk-mitigating practices between pig farmers in Denpasar and Karangasem.

Range score	Risk level	Denpasar		Karangasem		Total	
		
		Participants	Percentage	Participants	Percentage	Participants	Percentage
Score 0–4	Low risk	11	32.4	16	48.5	27	40.3
Score 5–10	High risk	23	67.6	17	51.5	40	59.7

## Discussion

Detection of the prevalence of HEV infection in pigs is important as the HEV infection of genotypes 3 and 4 is zoonotic, with pigs as the reservoirs of the HEV genotypes and the source of the viral transmission in the environment [17]. Although the infection in pigs is generally asymptomatic and subclinical, those infected animals are mainly responsible for human viral infection [18].

Assessment of the potential epidemic of zoonotic disease in humans requires related studies in its animal reservoirs. HEV is endemic in many Southeast Asian countries, especially in the areas where pigs are one of the important livestock for the communities [19]. The socio-ecological condition of the province of Bali, Indonesia, is mainly associated with pigs. Balinese tend to rear pigs for local ceremonies, consumption, and even investment [14]. However, these animals are generally reared in a traditional or extensive system, in which the hygiene and/or sanitation of the management rearing practices seems to be inadequate [20] and therefore, the risk of HEV exposure and transmission to humans is relatively high.

The total test seroprevalence obtained in this study was 23.5% (43/183), which is lower than the previous related studies on Bali Island conducted by Wibawa *et al*. [11] at 72% (51/71) and Wibawa *et al*. [16] at 57% (33/58). This individual animal-level seroprevalence result is even lower compared to the similar studies conducted by Utsumi *et al*. [21] and Widasari *et al*. [13], who assessed the prevalence of the antibodies against HEV in pigs sampled from Mengwi Subdistrict, Badung Regency, Bali at higher than 80% (n = 119). The lower seroprevalence obtained in this study might be related to a previous disease outbreak in pigs with high morbidity and mortality that occurred in Bali between 2020 and 2021 [22], which mostly impacted the whole pig population, including pork and related industries. Beginning in 2022, local pig farmers restarted rearing pigs with better rearing management practices, hygiene, disease prevention, and awareness. This “new” pig-rearing management is likely more effective in anticipating risk factors for infectious diseases in pigs.

Similar reasons may also apply to the herd-level seroprevalence obtained in this study, which is found to be at 36.8% (25/68). This level of seroprevalence was relatively lower compared to other studies. In non-endemic territories of the Russian Federation, more than 90% of HEV shedding was detected in 21 pig farms in the study areas [23]. This is in line with a study performed by Meester *et al*. [24], who outlined that the range of the farm-level seroprevalence of HEV in pigs with at least one pig seropositive sampled from the farm ranged from 60 to 100%.

The seroprevalence of the antibody to HEV in sampled pigs collected from Karangasem was higher compared to the pigs collected from Denpasar. Pigs collected from Karangasem were 5.3 times more likely to have the HEV antibody than those collected from Denpasar. This difference is likely associated with the husbandry or pig-rearing system being used to raise pigs. The farmer participants from Karangasem reared their pigs more traditionally or with an extensive management system (48.5%) than the farmers from Denpasar (32.4%). In the traditional pig-rearing system, herd or farm biosecurity seems less likely to be implemented, which may increase the risk of diseases or pathogens infecting the pigs [20]. A study conducted in Ghana found that there was a significant association between the seroprevalence of the HEV in pigs and husbandry or pig rearing systems (OR 7.05, 95% CI: 3.56–13.97, p < 0.001) and the region where pigs were collected (OR 4.6, 95% CI: 2.30–9.21, p < 0.001) [25].

In addition, the different seropositivity results obtained in the sampled pigs collected from Karangasem and Denpasar might also be associated with the results obtained from the survey in preventing the zoonotic diseases that can be transmitted from pigs to humans. A total of 58.8% of the participants from Denpasar reported that they prevented pig diseases that could be transmitted to humans, compared to only 30.3% of the participants from Karangasem. These results indicate that they had already been aware of pig diseases that could also be transmitted to humans and infect them. In association with the results obtained in this study, seroprevalence in pigs collected from rural areas in Karangasem Regency was higher than the seroprevalence in pigs collected from urban areas in Denpasar. This result is similar to the study conducted in China, where the HEV IgG level detected in humans in rural areas was higher (28.7%) than the level in those who were from urban areas (21.7%) [26].

This study showed that pigs over or equal to 6 months of age were 4 times more likely to be seropositive to HEV than pigs under 6 months of age. This may be because older pigs have a higher risk of being exposed to the virus and transmitted to them than younger ones. This result is similar to a study conducted in backyard pigs of north-eastern India, where pigs 7–12 months were found to have a HEV seroprevalence of 44.6% (78/175) (95% CI: 37.1–52.3), while pigs aged 13–24 months had 53.3% (48/90) (95% CI: 42.5–63.9), and pigs aged more than 24 months had 63.4% (26/41) (95% CI: 46.9–77.9). These outcomes indicate that the higher the age, the higher the risk of pigs being infected by the virus [27].

The results of this study also revealed that pigs reared in a traditional or extensive management system were 6 times more likely to be seropositive compared to those reared in a semi-intensive rearing system. In traditional systems, the cleanliness and/or hygiene of rearing the pigs and the pens does not seem to concern the farmers optimally. In this situation, the risk of the pigs being infected by pathogens, including HEV, also seems to be high. Non-sick and sick pigs are still placed in the same pen. Therefore, cross-HEV contamination among the pigs in a herd may accelerate the infection, contributing to the high HEV infection rate at the herd level. In Bali, piglets tend to be kept with older pigs, and as a consequence, direct and repeated contact among the pigs increases the risk of infection [13, 28].

The risk of pigs becoming infected by pathogens was also high in pigs that were reared in pigpens with non-cement floors (soil). The pathogens likely existed longer in conditions where the floors were moist. This situation makes the pathogens, including the HEV, likely to exist for a longer period of time before exposing and infecting other susceptible pigs in the pens. A study reveals a higher probability of HEV in swine slurries pig farming in the Abruzzo Region, where the pen floor tended to be moist [29]. This also leads to a higher risk for HEV spread and infection to other susceptible hosts around the herd, including humans in close contact with pigs.

In addition, some of the pigpens were observed to have accumulated or still had water on the floors of the participants’ pigpens (on the day when the survey was conducted). The water on the floor may cause the virus to exist for a longer period of time in the pen, leading to a higher risk for the susceptible pigs being exposed to and infected by the virus. Viremia HEV-infected pigs can shade and spread the virus through their excreta, including fecal and urinary shedding, which contaminate the pen floor, including the food and drinking water containers. This condition actually accelerates and exacerbates the infection in other susceptible HEV-infected pigs within the herd [24].

This study also showed that pigs reared in herds where a natural source of water existed nearby (within 300 m) were 6 times more likely to be seropositive compared to the pigs reared in herds with no natural water source in close proximity. In extensive pig-rearing systems, the pigs may be given drinking water that has originated from a natural source around the herds. That source of water may contain pathogenic substances, including viruses. In developing countries, HEV infection is also reported to be a waterborne illness that is linked to widespread outbreaks brought on by contaminated water and water supplies [30].

This study showed that seropositivity to HEV in pigs was detected, and it might indicate an association with the hepatitis cases in local humans as they tended to be in close contact with the animals. A study conducted by Koyuncu *et al*. [31] reported that, in general, humans who were in contact with animals had a 72% (n = 25) increased risk of being infected by HEV. In more specific terms, 32% of the risk of HEV-infected humans was from being in contact with animals at home and/or compounds, 24% in occupational exposures, and 16% from direct contact with animals in other settings.

This study also showed that pigs might have been infected by the HEV, indicating that local pig owners or farmers are at high risk of being exposed to and infected by the virus. There is an association between the high prevalence of antibodies in pigs and the high antibodies detected in pig farmers [32]. In addition, swine occupational exposure is associated with HEV infection, especially for workers in pig slaughterhouses, pork markets, or others who have a high risk of directly contacting infected pigs [3]. In consequence, this study suggests a survey of those communities for detecting their antibodies against the virus, including evaluating their knowledge, attitude, and practices regarding the risk of being exposed to and infected by the virus from coming into contact with the animals and their products.

This study’s results showed a relatively high herd-level prevalence of antibodies against HEV, indicating a high rate of viral infection among the sampled pigs, especially in the sampled pig herds of Karangasem. It seems that pig-rearing practices in the study areas need to be improved by increasing biosecurity systems and/or improving hygiene to lower the risk of infection. These strategies have already been suggested for lowering HEV infection in pig herds by improving pig farming practices in terms of hygiene, biosecurity, and rearing systems [9].

This study updates the recent survey data on the proportion of antibodies against HEV in pigs in the Province of Bali. To the best of our knowledge, it has been more than a decade since the last studies on HEV infection, especially in pigs, were updated. The previous studies that involved pig samples in Bali had reported with varied seroprevalence estimates, ranging from 57% (33/58) to 82.4% (98/119) [11, 13, 16, 21]. However, the present study found that the individual animal-level seroprevalence was lower compared to these previous studies. In fact, this study’s results show the first estimated herd-level seroprevalence in pigs on the island. However, the data presented in this study suggest that further surveillance should be performed, both in local Balinese and in animals, to predict the role of HEV transmission to humans in the study areas.

In this study, most of the farmers reported that they tended to wash their hands after going to pig herds and coming into contact with the animals. Hand washing leads to a lower risk of exposure and HEV transmission. This practice is believed to lower the risk of pathogen exposure and transmission from animals, as it was described in a study performed in Southwestern Nigeria, which found that the community of an animal contact group had a 2.2% higher incidence of HEV IgM compared to the non-animal contact group [33].

The pig farmers from Denpasar were more likely to engage in practices anticipating zoonotic disease, including washing their hands after going to pigpens, compared to the farmers from Karangasem. This may be because the farmers in the city had more access and information related to pig health management practices, including veterinary services. Such access and information are important to improve productivity and profitability outcomes [34]. In addition, communication and discussion among the local pig farmers, from the farmers to local veterinarians and animal health officers, and other related sectors in urban areas tend to be more intensive than those in rural areas. These frequent connections are beneficial for the health and productivity of the farmers’ livestock.

More than 50% of the total respondents reported that they had eaten undercooked pork along with their family members, putting them at high risk of viral exposure and transmission. HEV is a food-borne disease that can infect humans by eating undercooked pork of the infected pigs. In the USA, 12.6% (15/119) of ground pork samples and 45% (25/56) of pork liver samples tested positive for HEV RNA, indicating the pork products and the livers are likely the potential sources of the virus that could be transmitted to humans [35].

The results of this study indicate that the pig farmers are likely at high risk of being exposed to HEV and the virus transmitted to them as they come into contact with the pigs. In addition, all the farmers reportedly ate pork and its products. Some of them did eat raw or undercooked pork as well. Those who ate the undercooked pork were highly likely to be HEV exposed and the virus transmitted to them. In Belgium, pork liver pâtés and raw dried hams that were bought from the local supermarkets were 31% (17/54) detected to have HEV RNA and the isolates were phylogenetically similar to HEV-infected humans in the area [36]. In addition, a study conducted in Hebei, China, found that HEV RNA was detected in 6.1% (7/144) of pig livers, 3.1% (4/129) kidneys, and 1.2% (2/170) of the blood samples with viral loads ranging from 10^2.4^ to 10^4.4^ (2.4Log–4.4Log) genome equivalents per gram, indicating other organs or tissues of the infected pigs contained the virus and might also be the source of HEV infection in humans [37].

However, serological and virologic tests may be required to evaluate the proportion of HEV infection in local communities to investigate the risk and association between HEV infection in pigs and the infection in the community, especially in high-risk HEV-infected community groups. Other local occupational workers who have come into contact with pigs and pork products also need to be investigated, for instance, slaughterhouse workers and pork or meat vendors, to assess the risk of the pigs being the HEV source of infection in the communities. These surveys are required to demonstrate the route of HEV infection that has been suspected of spreading from the infected pigs to humans through direct contact with the pigs, HEV-contaminated environments, and the consumption of pork products that contain the virus [38].

The test kit used in this study did not provide sensitivity and specificity. Consequently, the real prevalence (RP) of HEV infection among pigs being tested could not be calculated. The RP of the samples tested is important to assess to estimate the real proportion of the antibodies detected from the sampled pigs.

Among the key finding obtained, this study only surveyed local pig farmers from two regions, Denpasar and Karangasem, which did not represent the whole of Bali. In addition, the sampling being performed in this study might also have certain biases that affect the results of the study. Therefore, further study with larger coverage areas and more participants is required to have better data on the risk level of local pig farmers toward HEV exposure and transmission in association with pig-rearing management practices.

## Conclusion

IgG antibodies against HEV were detected among pigs reared in rural areas of Karangasem and those reared in urban areas of Denpasar. This suggests that the strategy to control this potential zoonotic disease of HEV should not only focus on the rural areas but also on urban areas of Bali Province. Although, in some cases, the pig farmers from Denpasar more likely anticipated to being exposed to pathogens from animals and having the virus transmitted to them, all the pig farmers are categorized as potentially having a high risk of HEV exposure and transmission in relation to their pig rearing management practices. Public education on HEV infection, transmission, and prevention practices to anticipate HEV exposure and transmission need to be implemented, especially among local pig farmers, to minimize the risk of disease exposure and infection.

## Data Availability

The supplementary data can be available from the corresponding author on a reasonable request.

## Authors’ Contributions

IMK and APD: Conceptualized and designed the study and drafted and revised the manuscript. IMK, AAGOD, and IWNFG: Performed fieldwork study and collected samples. INMA, AAAMA, and PDJ: Conducted diagnostic test. IMK, IWNF, INSr, and INSt: Analysed and interpreted data. All authors have read, reviewed, and approved the final version of the manuscript.
